# A rare case of ascending colon adenocarcinoma incarcerated in an inguinoscrotal hernia: case report and literature review

**DOI:** 10.1186/s40792-018-0457-9

**Published:** 2018-05-21

**Authors:** Tien Yew Chern, Yeng Kwang Tay, Dayashan Shevantha Perera

**Affiliations:** Department of Colorectal Surgery, St George Hospital, 5/61 Port Hacking Road, Sylvania, NSW 2224 Australia

**Keywords:** Hernia, Inguinoscrotal hernia, Indirect hernia, Ascending colon cancer, Incarcerated cancer, Incarcerated adenocarcinoma, Incarcerated bowel cancer

## Abstract

**Background:**

Inguinal hernias and colorectal cancers are common conditions, but the presentation of a loop of bowel containing cancer within a hernia is rare. Principles of surgery include oncological resection of the involved colonic segment as well as lymphatic drainage. Based on case reports of the last several decades, there have been no reports of a case where the reduction of an inguinoscrotal hernia and oncological colectomy were performed completely laparoscopically. We present the first instance of a completely laparoscopically assisted resection and hernia repair on a patient with T4 ascending colon cancer. A literature search on recent case reports over the last 30 years has also been presented with a focus on trends in treatment.

**Case presentation:**

An 83-year-old man presented for further investigation of his iron deficiency anaemia and was diagnosed with adenocarcinoma of the ascending colon. This was demonstrated radiologically to be found within a large right inguinoscrotal hernia. He underwent a laparoscopically assisted right hemicolectomy and laparoscopic closure of the internal ring and recovered well.

**Conclusions:**

Colorectal cancers within inguinal hernias are rare and can often present with complications such as perforation. As such, treatment has mostly involved an open operation. The last few years have shown feasibility of a laparoscopic approach and can be attempted safely when indicated.

## Background

The combination of inguinal hernia and colorectal cancer is a very uncommon presentation with less than 40 reports found in the literature. Given its rarity, there has been no standard treatment and therefore often presents to the treating team a therapeutic dilemma. We present a rare case of inguinoscrotal hernia containing an ascending colon adenocarcinoma presenting initially as anaemia for further investigation and eventually treated completely laparoscopically. Given the variability in treatment in the literature and the increasing use of laparoscopic techniques since its introduction in the 1990s, we have also included a literature review with a focus on trends in treatment over the last 30 years.

## Case presentation

**Fig. 1:**
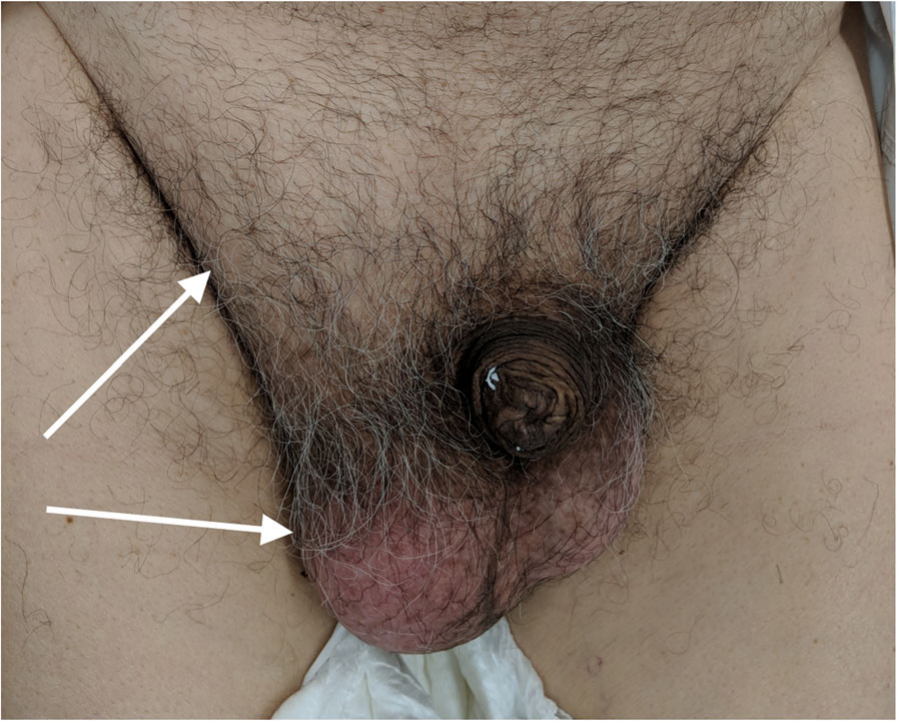
Pre-operative inguinoscrotal hernia. White arrows point to the right-sided inguinoscrotal hernia

An 83-year-old man on clopidogrel for coronary artery disease presented to the emergency department of our institution with symptoms of anaemia for the past 3 weeks (Table 1) including increasing fatigue and increasing shortness of breath on exertion. He denied any per rectal bleeding or change in bowel habits and had no significant weight loss. He was noted to have a large partially reducible right inguinoscrotal hernia on examination containing bowel (Fig. 1) which he stated had been present for more than 10 years, which had remained unchanged in size in the last few years.

**Table 1:** Pre-operative blood results

Blood results	
Sodium	140 mmol/L
Potassium	5.1 mmol/L
Haemoglobin	74 g/L
Haematocrit	0.244 L/L
Mean cell volume	6.6 fL
Mean cell haemoglobin	21.2 pg
Mean cell haemoglobin concentration	303 g/L
Red cell distribution width	37.2 fL
Iron	3.2 μmol/L
Iron saturation	3.8%
Transferrin	3.4 g/L
Ferritin	4 μg/L
Carcinoembryonic antigen	2 μg/L

His other significant background consisted of hypertension, three previous transient ischaemic attacks, type 2 diabetes mellitus and had a permanent pacemaker for sick sinus syndrome.

Colonoscopy demonstrated a large non-obstructing tumour located at the ascending colon. The tumour was confirmed as adenocarcinoma on histopathology.

**Fig. 2:**
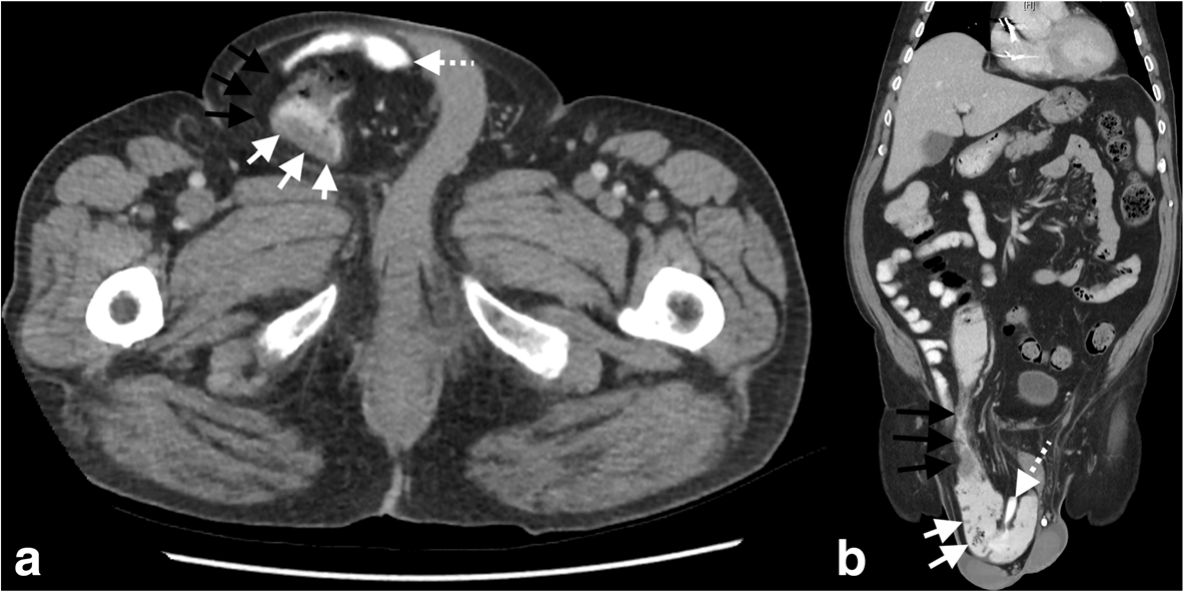
Computed tomography. **a** Axial view demonstrating right-sided inguinoscrotal hernia (black arrows) containing large bowel with thickening of the wall representing the ascending colon cancer (white arrows) and herniated terminal ileum (dashed white arrow). **b** Coronal view demonstrating the right-sided inguinoscrotal hernia (black arrows) with large bowel (white arrows) and terminal ileum (dashed white arrow) herniating through the inguinal canal

Further investigations included a CEA level of 2 μg/L and computed tomography of the chest and abdomen which did not demonstrate metastatic disease but that the ascending colon tumour was located in his right inguinoscrotal hernia (Fig. 2).

**Fig. 3:**
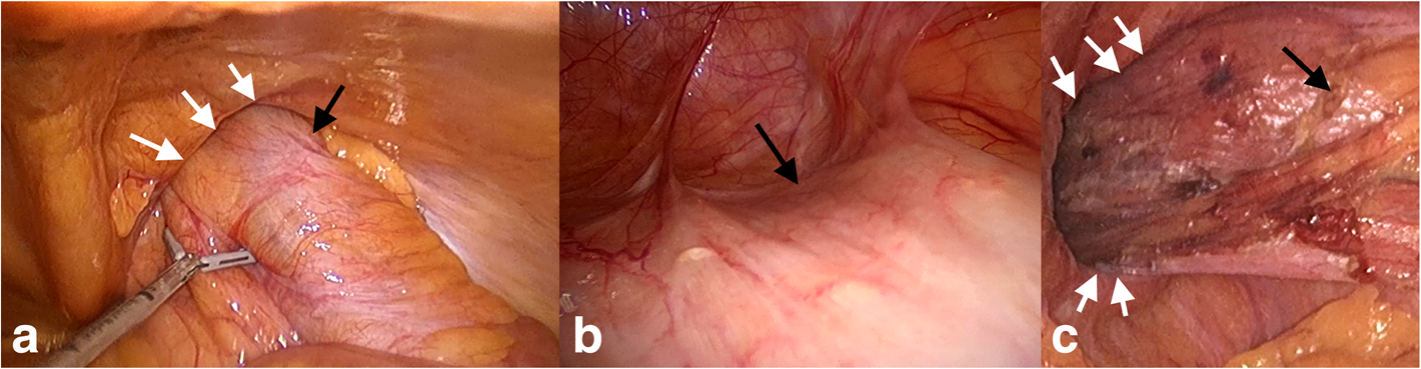
Intra-operative images. **a** Laparoscopic view of ascending colon (black arrows) herniating through the deep ring (white arrows). **b** Angled view within the hernial sac (inguinal canal) using the 30° laparoscope, black arrows pointing to adherent ascending colon. **c** Laparoscopic view of the deep ring (white arrows) with ascending colon (black arrows) and terminal ileum reduced

He underwent a laparoscopic right hemicolectomy. The caecal pole was adherent to the scrotal sac, and the adhesion was deemed to be inflammatory related rather than direct invasion from the tumour. Laparoscopic adhesiolysis was performed with a moderate degree of difficulty. A 30° laparoscope was used and inserted into the inguinoscrotal hernia for an angled view (Fig. 3). Bowel manipulation was performed with atraumatic graspers, and adhesiolysis was achieved by an energy device (Olympus Thunderbeat™). Further mobilisation followed a standard lateral to medial approach, followed by inferior to superior mobilisation. The omentum was dissected off the transverse colon, lesser sac entered and hepatic flexure to transverse colon was mobilised. The ileocolic pedicle was isolated and stapled, and the diseased colon was delivered through the umbilical port site. A resection and a functional end-to-end anastomosis were performed with the GIA™ 80 mm stapler (Medtronic). Pneumoperitoneum was then re-established, and the deep ring was closed snugly around cord structures with 2-0 V-Loc™ (Medtronic) non-absorbable sutures laparoscopically.

He had an unremarkable post-op recovery and was discharged home after 7 days. There was no recurrence of the inguinoscrotal hernia at his 4-week post-operative review.

Histopathology confirmed that the ascending colon mucinous adenocarcinoma had invaded partially into serosa with clear resection margins. Four out of 15 lymph nodes were positive. TNM staging was T4aN2aM0 with an R0 resection.

Given the high-risk cancer and poor prognostic features, he is currently being planned for adjuvant capecitabine monotherapy.

## Discussion

The incidence of inguinal hernias in the western population is high with an incidence of 1.7% with men accounting for 95% of the presentations [1]. In addition, incidence of colon cancer in the western population is 40 per 100,000 and is currently the fourth leading cause of cancer mortality worldwide [2]. However, together, they form a rare entity with only 38 cases (see Appendix 1) with primary colorectal cancer being reported in the literature worldwide, with all but one cases occurring in males who are mostly elderly (mean age = 71.7 years) [3, 4]. Gerhardt in 1938 was the first to report this entity involving the sigmoid colon, and since then most reporters (71.1%) have documented sigmoid involvement. Caecal cancers are the second most likely (15.8%), followed by ascending colon cancers, of which there have been two (5.2%) including this case report. Due to the rarity of this entity, there has been no clear guideline for treating this.

Therefore, and in the context of advances in surgical treatment especially with the introduction of laparoscopically assisted colectomies since the 1990s, we present a literature review of cases over the last 30 years (Table 2). While there has been little difference in mean ages (71.3 years in the earlier group and 72.1 years in the later group), we have found that these patients tend to present as complicated hernias with 8 of 17 (47.1%) being incarcerated, followed by perforation (41.2%), 1 strangulation (5.9%) and 1 uncomplicated hernia (5.9%).

**Table 2:** Cases of bowel cancer presenting within inguinal hernias in the last 30 years

Year	First Author	No	Sex	Age	Site	Complications	Treatment	Incision	LOS (days)
1991 [13]	Hale	22	M	85	Sigmoid	Incarceration	Hartmann’s + intra-abdominal internal ring closure	Midline laparotomy	NA
2003 [12]	Tan	23	M	62	Sigmoid	Incarceration	High anterior resection + left orchidectomy + intra-abdominal closure of hernial defect	Left iliac fossa transverse	NA
2003 [7]	Kouraklis	24	M	79	Sigmoid	Perforation	Hartmann’s + loop colostomy + interval reversal and open hernioplasty	Left groin	10
2003 [16]	Cervinka	25	M	69	Sigmoid	Incarceration	Sigmoidectomy + anastomosis + defunctioning transverse colostomy + herniorrhaphy (Bassini)	Left groin	NA
2008 [17]	Slater	26	M	66	Sigmoid	Incarceration	Hartmann’s + left orchidectomy + internal ring closure	Left groin + midline laparotomy	NA
2008 [17]	Slater	27	M	73	Sigmoid	Perforation	Hartmann’s + left orchidectomy + internal ring closure	L groin + Left modified Rutherford-Morrison	NA
2009 [18]	Ruiz-Tovar	28	M	67	Sigmoid	Perforation without contamination	Sigmoidectomy and anastomosis + hernioplasty (Lichtenstein)	Left groin + midline laparotomy	7
2010 [19]	Ko	29	M	84	Sigmoid	Perforation	Hartmann’s + herniorrhaphy	Laparotomy	Deceased
2010 [20]	Mai	30	M	83	Sigmoid	Strangulation	Hartmann’s + herniorrhaphy	Lower midline + left groin	NA
2011 [5]	Pernazza	31	M	70	Caecum	Incarceration	Laparoscopic right hemicolectomy + herniorrhaphy	Laparoscopy + bilateral groin incisions	5
2012 [6]	Carr	32	M	65	Sigmoid	Incarceration	Laparoscopic high anterior resection + open biosynthetic hernioplasty	Laparoscopy + left groin	4
2013 [21]	Meniconi	33	F	78	Caecum	Incarceration	Open right hemicolectomy + herniorrhaphy	Midline laparotomy + right groin	NA
2013 [22]	Tan	34	M	63	Sigmoid	Perforation	Hartmann’s + herniorrhaphy	Midline laparotomy + left groin	10
2016 [23]	Kulasegaran	35	M	66	Sigmoid	Perforation + necrotising infection	Hartmann’s + debridement + staged skin graft	Left groin + midline laparotomy	NA
2016 [24]	Diao	36	M	48	Transverse colon	Perforation	Transverse colectomy + herniorrhaphy + orchidectomy + partial scrotectomy	Left groin	NA
2017 [25]	Sharma	37	M	84	Caecum	None	Open right hemicolectomy + herniorrhaphy	Right transverse	NA
2018	Current report	38	M	83	Ascending colon	Incarceration	Laparoscopic right hemicolectomy + laparoscopic defect closure	Laparoscopy	10

*LOS* length of stay

Most patients undergoing the procedure had two incisions, one being the initial incision at the hernia site discovering the pathology followed by a midline incision for the oncological resection. Given the high rates of perforation, eight patients (47.1%) had an end colostomy (Hartmann’s operation). Other than this reported case, there have been two attempts at laparoscopic repair, both in incarcerated but non-perforated hernias, with Pernazza et al. [5] performing a laparoscopic right hemicolectomy and Carr and O’Dair [6] performing a laparoscopic anterior resection.

Most reports document performing the hernia repairs in the same operation although Kouraklis et al. [7] who had formed a loop colostomy delayed that operation for 6 weeks and performed a mesh repair during the colostomy reversal operation. Citing fears of contamination and risk of mesh infection, 11 reporters (64.7%) have chosen to perform open non-mesh repairs with 2 of these being intra-abdominal defect closures. Of the three mesh repairs, all have been performed in cases without significant risk of sepsis, although there was perforation without contamination in the case reported by Ruiz-Tovar et al.; nonetheless, there were no reported complications. None of the cases had hernias repaired laparoscopically.

Our case is the second known case of inguinoscrotal hernia containing an ascending colon cancer and to our knowledge the first case in which both the bowel resection and hernia repair were performed completely laparoscopically. The decision to perform the procedure laparoscopically was based on several factors.

1. The benefits of laparoscopic right hemicolectomy are well known, and two recent meta-analyses [8, 9] of non-emergent laparoscopic vs open right hemicolectomies have demonstrated advantages intraoperatively with reduced blood loss, post-operatively with reduced time to first flatus, reduced length of stay, reduced post-operative pain, reduced rate of wound infections with oncologically similar results, with similar number of lymph nodes taken, length of resection and rates of recurrence. The only disadvantage has been increased operative time.
2. While our patient presented to the emergency department, the procedure was carried out electively and was initially investigated endoscopically and thence underwent radiological and biochemical investigations allowing for planning and discussion of the planned elective procedure. Given the benefits of laparoscopic right hemicolectomy, we felt that it was in the patient’s best interest to proceed laparoscopically with an option to convert to open if required. This was discussed with the patient who agreed and consented to our approach.
3. Given the unusual presentation of the cancer, laparoscopy allowed definition of the disease with minimal trauma and allowed the flexibility of treatment to change if required without committing to a surgical strategy dictated by an open incision.
4. Although bowel was incarcerated within the hernia, the defect was thought to be large given the fact that the colonoscope was able to reach the terminal ileum and hence passing through the inguinal canal within bowel. As such, muscle relaxation and pneumoperitoneum might have allowed reduction of the herniated bowel which was previously non-reducible [5].

Given the risk of herniation through the large defect, it was also important that the hernial defect was repaired. With our centre’s long experience with laparoscopic hernioplasties, a trans-abdominal preperitoneal (TAPP) approach was considered. As the operation entailed a bowel resection, the wound created was considered clean contaminated, and despite significant reduction in wound infection rates in laparoscopic right hemicolectomies, they remain at 5.3% [9]. Therefore, a TAPP approach was thought to be unfeasible given the risk of a surgical site infection and that the only barrier between mesh and bowel was dissected peritoneum, putting the mesh at risk of contamination and infection potentially resulting in a difficult re-operation to remove the mesh.

An open approach and tissue repair was also considered with the advantage of being able to excise the sac for histopathological analysis although the benefits remain unclear in primary intrasaccular tumours [3]. Given the benefits of laparoscopic hernioplasties with chronic pain [10, 11], it was decided that the hernial defect could feasibly be closed laparoscopically, noting that previous approaches also included open but intra-abdominal closures of the hernial defect or internal ring [12, 13]. This seemed to be a good compromise, and should the hernia recur, the patient could have a laparoscopic hernioplasty electively.

While it has been established that there is no benefit to screening for abdominal cancers endoscopically or radiologically in inguinal hernias [14], any patient presenting with suspicious features such as symptoms of anaemia, per rectal bleeding, weight loss and change in bowel habits should be further investigated.

It has been traditionally advocated that T4 tumours are to be resected using an open approach. Recent data have challenged that and have shown that a laparoscopic approach can be safe and is not significantly inferior to an open approach in terms of oncologic outcomes and at the same time maintaining the key advantages of laparoscopy as previously described [15].

## Conclusions

An inguinal hernia containing colorectal cancer is rare but serious and can often present with complications such as perforation. We have presented a case of T4 ascending colon cancer within an inguinoscrotal hernia treated completely laparoscopically. Although the data are limited, laparoscopically assisted bowel resections in this context can be safely performed and could be attempted if feasible.

## Appendix 1

**Table 3:** All cases of bowel cancer presenting within inguinal hernias (adapted from Matsumoto et al. [3] and Slater et al. [17])

No	Sex	Age	Site	First author	Year
1	M	54	Sigmoid	Gerhardt	1938
2	M	60	Sigmoid	Fieber	1955
3	M	66	Sigmoid	Bruce	1958
4	M	67	Sigmoid	Lookanoff	1960
5	M	74	Sigmoid	Griffiths	1964
6	M	68	Sigmoid	Lees	1966
7	M	62	Caecum	Silberman	1969
8	M	76	Caecum	Dross	1973
9	M	73	Sigmoid	Dross	1973
10	Unknown		Colon	Dross	1973
11	Unknown		Colon	Dross	1973
12	M	71	Sigmoid	Horvath	1974
13	M	86	Ascending colon	Gross	1980
14	M	73	Sigmoid	Gross	1980
15	M	70	Sigmoid	Kanzer	1983
16	M	63	Sigmoid	Siriam	1986
17	M	85	Caecum	Siriam	1986
18	M	80	Sigmoid	Papas	1987
19	M	86	Sigmoid	Lafferty	1989
20	M	75	Sigmoid	Lafferty	1989
21	M	66	Sigmoid	Lafferty	1989
22	M	85	Sigmoid	Hale	1991 [13]
23	M	62	Sigmoid	Tan	2003 [12]
24	M	79	Sigmoid	Kouraklis	2003 [7]
25	M	69	Sigmoid	Cervinka	2003 [16]
26	M	66	Sigmoid	Slater	2008 [17]
27	M	73	Sigmoid	Slater	2008 [17]
28	M	67	Sigmoid	Ruiz-Tovar	2009 [18]
29	M	84	Sigmoid	Ko	2010 [19]
30	M	83	Sigmoid	Mai	2010 [20]
31	M	70	Caecum	Pernazza	2011 [5]
32	M	65	Sigmoid	Carr	2012 [6]
33	F	78	Caecum	Meniconi	2013 [21]
34	M	63	Sigmoid	Tan	2013 [22]
35	M	66	Sigmoid	Kulasegaran	2016 [23]
36	M	48	Transverse colon	Diao	2016 [24]
37	M	84	Caecum	Sharma	2017 [25]
38	M	83	Ascending colon	Current report	2018

## Abbreviations

LOS: Length of stay
TAPP: Trans-abdominal preperitoneal approach
